# A very interesting traditional method in the treatment of skin lesions: aqua regia and related complications

**DOI:** 10.1186/s40064-016-3259-1

**Published:** 2016-09-15

**Authors:** Fatih Doğan, Cemal Alper Kemaloğlu

**Affiliations:** 1 Department of Plastic, Reconstructive and Aesthetic Surgery, Erciyes University, Kayseri, Turkey; 2 Department of Plastic, Reconstructive and Aesthetic Surgery, Adıyaman University, Altınşehir Mh. 3005 Sokak No:13, 02040 Adıyaman, Turkey

**Keywords:** Aqua regia, Chemical burns, Non-medical treatments

## Abstract

**Background:**

Aqua regia, a highly corrosive mixture of concentrated nitric acid and hydrochloric acid, is used to dissolve various metals such as gold and platinum that are not soluble in other types of acids. For the first time in the literature, we report the adverse effects that were observed following the utilization of this strong acid solution in the treatment of various skin lesions.

**Methods:**

Between 2010 and 2013, 43 patients (55.8 % female) with a mean age of 40.2 years, were admitted to our hospital for scars, wounds or malign lesions related to the use of aqua regia on skin lesions. Sixteen patients reported to use aqua regia for treating wounds and 27 of them used it for their scars.

**Results:**

Out of 43 patients, 9 patients developed necrosis, 12 had hypertrophic scars, 15 had scars and 7 had non-healing wounds resembling malignant lesions. The non-healing wounds were located in the facial region in five patients, in the cervical region in one and on the finger-tip in another. Histopathological examination of these non-healing wounds revealed basal and squamous cell carcinoma. The patients were admitted to the plastic surgery department approximately 6–24 months after aqua regia application with complaints of growing lesions. Secondary healing in the lesions with scar development in 55 % of the patients is an acceptable result. However, 27 % of the patients with hypertrophic scars and 16 % of the patients with malignant lesions required intervention.

**Conclusion:**

This present study showed that non-medical alternative treatments have major risks. Aqua regia application might have resulted in the development or rapid progression of malignant tumours in seven patients. In the literature, it has been reported that a number of physical traumas may cause skin cancer. Based on this information, the possibility of such an effect of aqua regia cannot be excluded.

## Background

Although modern medicine can cure most of the diseases, attraction to alternative medicine can be seen in every culture. Many patients with dermatological problems try one or another form of complementary/alternative medicine. Survey data suggest that lifetime prevalence of complementary/alternative medicine use by dermatological patients range from 35 to 69 % (Ernst 2000a, b). The reasons for this increasing popularity of complementary/alternative medicine are complex but partly related to the fact that patients are being bombarded by the popular media with the message that complementary/alternative medicine is effective and largely devoid of the adverse effects of orthodox treatments. Although there are reports on the undesired outcomes of alternative treatments in Turkey (Eskitascioglu et al. 2008; Yenidünya et al. 1999), to our knowledge, there is no report on aqua regia application on skin lesions in the literature. Patients reported in this present study had used this chemical agent as an alternative method for the treatment of their skin lesions, and were admitted to our hospital due to the complications they experienced.

Aqua regia or nitro-hydrochloric acid is a highly corrosive mixture of acids. It is the mixture of concentrated nitric acid and hydrochloric acid, usually mixed in a volume ratio of 1:3 (Low and Bansal 2010). This solution is used to dissolve various metals such as gold and platinum that are not soluble in other types of acids, for the analysis of minerals and, and cleaning of metal surfaces. There is no report in the literature on the use of aqua regia in diseases, nor on the injuries caused by aqua regia.

It was mentioned by several patients that the people working at one of the jewellery stores in the neighbourhood used this solution as an alternative non-medical treatment for treating skin lesions. For the first time in the literature, we report the undesired conditions that were observed following the utilization of this strong acid solution in the treatment of various skin lesions.

## Patients and methods

Between 2010 and 2013, 43 patients (55.8 % female) were admitted to the clinics of our hospital for scars, wounds or malign lesions related to the use of aqua regia on skin lesions. The mean age of the patients was 40.2 years (range 11–78 years). Sixteen of the patients reported that they had used aqua regia for treating wounds and 27 of them stated that they had used it for their scars. The lesions were located in the facial region in 39 cases, in the cervical region in three cases and on the fifth finger of hand in one case.

The patients stated that they used this alternative medicine by recommendation of other people. Aqua regia was applied to the skin lesion using a non-sterile stick with cotton tip by the people working at the jewellery store. After the application of aqua regia, the lesions turned white and subsequently they were crusted and fall off. The patients were admitted to the hospital because of the development of non-healing wounds or scars after the application of aqua regia.

## Results

Out of 43 patients, 9 patients developed necrosis, 12 had hypertrophic scars, 15 had scars and 7 suffered from non-healing wounds resembling malignant lesions. The non-healing wounds were located in the facial region in five patients, in the cervical region in one and on the finger-tip in another (Table 1). Histopathological examination of the biopsies obtained from these non-healing wounds revealed basal and squamous cell carcinoma. Upon the recommendations of other people, these patients sought advice from the people working at the jewellery store for their skin lesions, instead of consulting a physician. The patients were admitted to the plastic surgery department approximately 6–24 months after aqua regia application with complaints of growing lesion. Secondary healing of the lesions with scar development in 55 % of the patients was an acceptable result. However, 27 % of the patients with hypertrophic scars and 16 % of the patients with malignant lesions required intervention.

**Table 1:** Locations and histopathologic examination of complications related with aqua regia application

Complications	Locations		
	Facial	Cervical	Finger
BCC	4	1	
SCC	1		1
Skin necrosis	9		
Hipertrofic scar	10	2	
Scar	15		

### Sample cases

#### Case #1

**Fig. 1:**
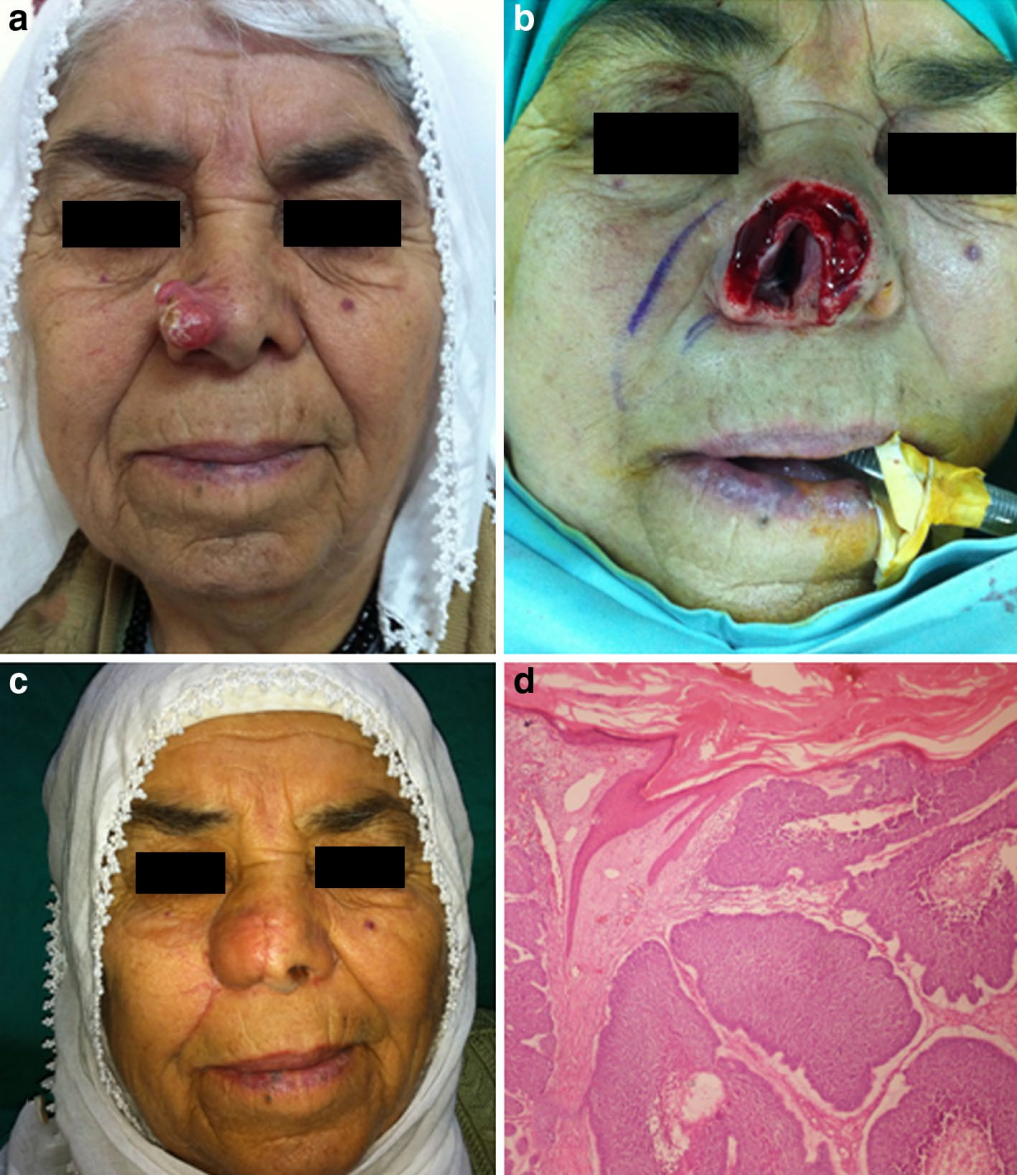
Nasal basal cell carcinoma. **a** The lesion had been growing rapidly for the last 6 months, **b** intraoperative appearance: Tumour was infiltrated the nasal mucosa. **c** The defect was reconstructed with nasolabial flap. **d** Histopathological examination of the lesion (Hematoxylin-EosinX10)

A 68-year-old female patient with a papular lesion on the right side of the nose for a year was admitted to our clinic complaining that the lesion had been growing rapidly for the last 6 months. Aqua regia had been applied to the lesion 6 months after the appearance of the lesion. Thereafter, the lesion showed rapid progression for an additional 6 months and the tumour infiltrated the nasal mucosa. She underwent surgery including the excision of the right nasal ala and reconstruction with a nasolabial flap under general anaesthesia. In the histopathological examination, the lesion was diagnosed as basal cell carcinoma. The patient is being followed up for 2 years with no evidence of recurrence (Fig. 1).

#### Case #2

**Fig. 2:**
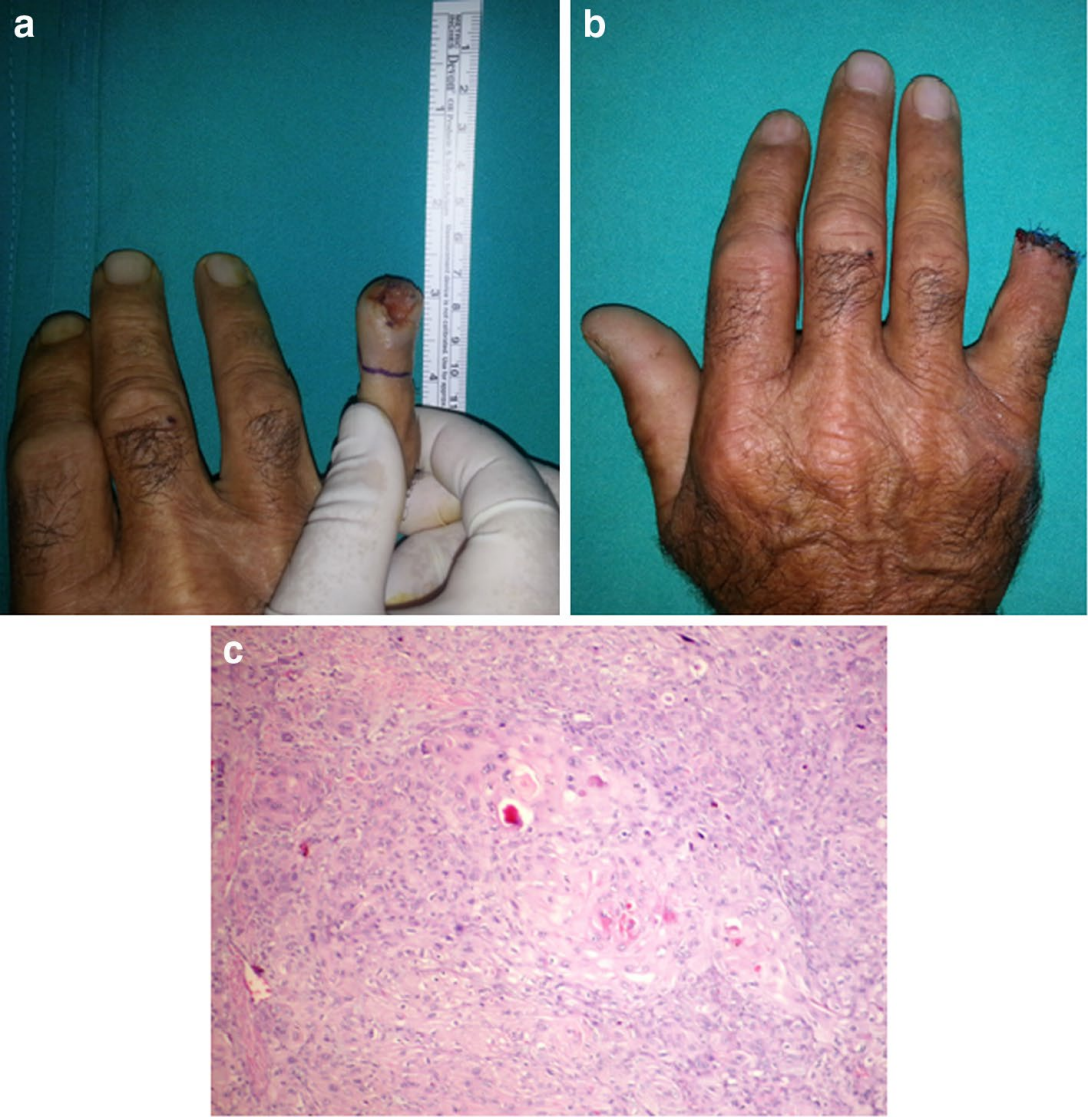
Fingertip squamous cell carcinoma. **a** Appearance of the lesion. **b** Surgical amputation was performed for bone invasion. **c** Histopathological examination of the lesion (Hematoxylin-EosinX10)

A 65-year-old male patient, who had squamous cell carcinoma on the distal part of the fifth finger, was admitted to our hospital 6 months after aqua regia application. Surgical amputation was performed for bone invasion. No recurrence was observed in the 1 year follow-up period (Fig. 2).

#### Case #3

**Fig. 3:**
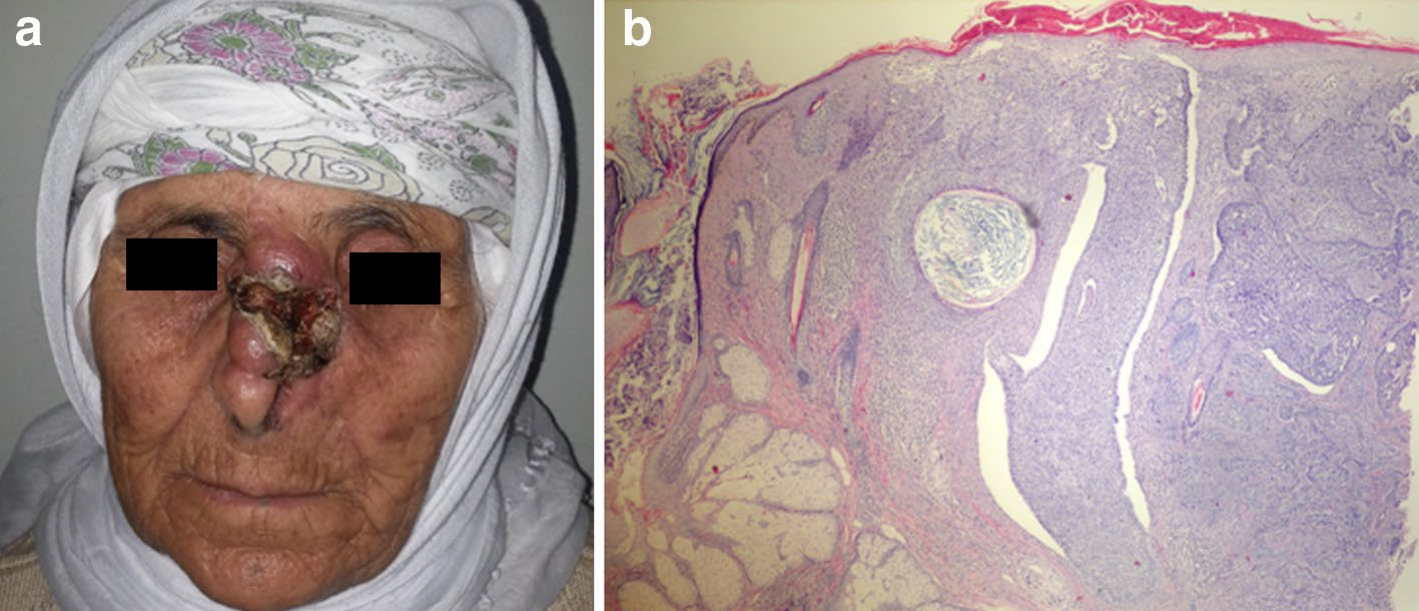
Nasal basal cell carcinoma. **a** The lesion had been growing rapidly after application of aqua regia for 2 years. **b** Histopathological examination of the lesion (Hematoxylin-EosinX4)

A 78-year-old female patient with a nodular lesion on the dorsum of the nose for 3 years was admitted to our hospital. Aqua regia was applied to the lesion after 1 year of the development of the lesion. She stated that the lesion had been growing rapidly since then. An incisional biopsy was performed. In the histopathologic examination, the lesion was diagnosed as basal cell carcinoma (Fig. 3). The patient did not accept surgical intervention.

## Discussion

Although modern medicine can treat most of the diseases, tendency to alternative treatment methods may be seen in every population. Alternative treatments, used as an addition to conventional medicine or instead of them, has been increasingly used recently (Ernst 2000a, b). Possible reasons for this increase may be the easy access of patients to complementary and alternative treatment products, the common belief in society that alternative medicine is efficacious, unmet health requirements and sociocultural characteristics (Zollman and Vickers 1999). Commercials on complementary/alternative treatments in mass media, inadequate information on the products and methods, and insufficient inspection of the people or the places of application lead patients to prefer such applications. Studies concerning social knowledge, and the usage level of complementary and alternative medicine in Turkey are quite limited, except for the information on alternative medications used in cancer and rheumatic diseases (Gözüm et al. 2003). Although there are reports on the undesired outcomes of alternative treatments (Eskitascioglu et al. 2008; Yenidünya et al. 1999), to our knowledge, there is no report in the literature on the application of aqua regia on skin lesions. The patients reported in this study had used this chemical agent as an alternative method for the treatment of their skin lesions, and they were admitted to the clinics of our hospital due to complications they experienced.

Chemical agents cause protein denaturation in tissues. Biological proteins are three-dimensional structures made up of amino acid sequences and weak intermolecular bonds such as hydrogen and van der Waals bonds. The three dimensional structure providing biological activity to the proteins may easily be impaired by external factors including heat, chemical agents or alterations in pH may lead to the deformation of these bonds and result in protein denaturation. Aqua regia, composed of hydrochloric acid and nitric acid, is a strong acid solution with corrosive and oxidative properties. Both acids cause tissue damage by a reduction reaction (Arthur et al. 2007). These acids act on the bonds of tissue proteins via free electrons and subsequently result in protein denaturation. In this study, the use of aqua regia caused necrosis by protein denaturation and scars were formed due to secondary wound-healing.

Malignant skin lesions were diagnosed in seven of the patients, and it could not be clearly understood whether these lesions were benign or malignant in nature before aqua regia application. However, the rapid progression of the tumours following the application is remarkable. A squamous cell carcinoma case due to hydrochloric acid injury was reported in the literature (Kargi et al. 1999). In this patient, the carcinoma was observed 4 months after the injury. Similarly, the patients in our study were admitted to our hospital approximately 6 months following the application of aqua regia. The patients reported that they did not feel pain during application and the application induced a rapid necrosis in the early period. The non-compliance of the patients with the treatment and follow-up may give a clue to the reasons for their preferring this non-medical application.

Alternative treatments have been increasingly used among the general population, worldwide. The prevalence of complementary/alternative medicine use range from 9 to 65 % (Ernst 2000a, b). In fact, destructive-ablative treatments such as freezing, curettage-electrodesiccation or laser application have a place in the non-surgical treatment of skin cancer (Kokoszka and Scheinfeld 2003; Goldman 2002; Marmur et al. 2004). However, they are not considered as the primary treatment of skin cancer due to the high rate of recurrence and resulting deformities (Dubin and Kopf 1983; Bae et al. 2005; Jung et al. 2011). In this article, the elimination of the malignant and benign lesions by non-medical aqua regia application is not surprising. Acids can eliminate the primary lesion by protein denaturation and cell death. However, this present study showed that non-medical alternative treatments have major risks. One of them was that the patients were exposed to a chemical agent, and more importantly, it was not possible to make a histopathological diagnosis of the primary lesion. Since the lesion was not examined before aqua regia application treatment and follow-up was delayed. This non-medical alternative method might have led to the development or rapid progression of malignant tumours in seven patients. In the literature, it has been reported that a number of physical traumas may cause skin cancer (Ozyazgan and Kontas 2004; Noodleman and Pollack 1986; Kandamany and Monk 2009). Based on this information, the possibility of such an effect of aqua regia cannot be excluded completely.

## Conclusion

This present study showed that non-medical alternative treatments have major risks. The patients were exposed to chemical agents, and more importantly histopathological diagnosis of the primary lesion was impossible. Since the lesions were not examined before aqua regia application, there was a delay in treatment. Aqua regia application might have resulted in the development or rapid progression of malignant tumours in seven patients. In the literature, it has been reported that a number of physical traumas may cause skin cancer. Based on this information, the possibility of such an effect of aqua regia cannot be excluded.
